# Diverse microbial communities colonize biofilm carriers in moving bed and intermittent cleaning reactors for municipal and industrial wastewater treatment

**DOI:** 10.1016/j.bioflm.2025.100337

**Published:** 2025-11-20

**Authors:** Eshetu Janka, Ram Prasath Alagappan, Dipaluk Das, Leif Arne Kjeldsberg, Shuai Wang, Tone Haugen, Alexander Wentzel

**Affiliations:** a Department of Energy and Environmental Technology, University of South-Eastern Norway, Kjølnes ring 56, 3918, Porsgrunn, Norway; b Biowater Technology AS, Grev Wedels gate 1, Tønsberg, Norway; c SINTEF Industry, Department of Biotechnology and Nanomedicine, Torgarden, N-7465, Trondheim, Norway

**Keywords:** Bacteria, Biofilm, Biofilm carrier, Surface area, Diversity, Wastewater, Footprint, MBBR, CFIC

## Abstract

Conventional biofilm reactors are widely used for the removal of organic constituents and nutrients (i.e., nitrogen and phosphorus) in municipal and industrial wastewater treatment. Moving bed biofilm reactor (MBBR) and continuously flow intermittent cleaning reactor (CFIC) have been developed as more compact, small footprint, and highly efficient biofilm-based systems for wastewater treatment. However, despite the advancements in reactor technology, there is limited scientific information on the microbial composition of the biofilms in these systems. This study aimed to characterize and provide early insights into the microbial diversity of biofilms grown on biofilm carriers and liquid suspensions of the biofilm-based wastewater treatment systems. Microbial samples were collected from biofilm carriers and liquid suspension in four full-scale MBBR plants and two CFIC plants in Norway, all treating municipal and industrial wastewater. DNA was extracted from the samples and subjected to *meta*-barcode sequencing for taxonomic classification of microbial communities in each treatment plant. The results revealed significant variation in microbial compositions across treatment plants, influenced by wastewater characteristics, biofilm carrier types, and reactor operational characteristics. On the biofilm carriers, the dominant bacterial taxa included TM7-1 (*Saccharibacteria*), *Burkholderiales*, *Clostridiales*, *Actinomycetales*, *Pseudomonadales*, *Rickettsiales*, and *Rhodobacteriales*. In liquid suspensions, the dominant groups were *Clostridiales*, *Methanosarcinales*, *Pseudomonadales*, *Flavobacteriales*, and *Rhodobacteriales*. In conclusion, this study highlights the diverse microbial populations on biofilm carriers and liquid suspensions, which collectively contribute to the enhanced treatment efficiency of both MBBR and CFIC systems.

## 1 Introduction

Conventional aerobic biological wastewater treatments are primarily designed to oxidize biodegradable constituents, incorporate solids into biological flocs or biofilms, and remove nutrients (i.e. nitrogen and phosphorus) and specific trace organics [[1], [2], [3]]. These treatments operate as either suspended growth or an attached growth process. In a suspended growth process (e.g. activated sludge processes), bacteria grow in liquid suspension. A secondary clarifier is typically used to settle and separate the biomass from the treated effluent. In contrast, attached growth processes involve microbes adhering to surfaces, such as filtration membranes in membrane bioreactors (i.e., MBRs) or mobile carriers in moving bed biofilm reactors (i.e., MBBRs), which are not fixed to the reactor surface [[4], [5], [6]]. Both systems achieve nutrient removal through various biochemical reactions. These include aerobic biodegradation of organics by heterotrophic bacteria, nitrifiers, and phosphorus accumulating organisms (PAOs), as well as anoxic processes facilitated by denitrifiers and anaerobic ammonium oxidizers (i.e., anammox) [2,7,8].

Over the past few decades, emerging technologies have significantly enhanced wastewater treatment performance. One such innovation is the moving bed biofilm reactor (MBBR), developed in Norway in the early 1990s for small community plants with a capacity of 2000 PE (population Equivalent) [5,9]. Since then, MBBR technology has been widely adopted, enabling cost-effective upgrades of larger treatment plants. Its popularity stems from advantages such as reduced sludge production, minimal clogging, a compact footprint, and biofilms that are more resilient to variations in influent characteristics, including shock loads, pH, temperature, and toxic compounds [5,9]. Today, over 1200 wastewater treatment plants in more than 50 countries use MBBR technology [5,10]. Building on its foundational principles, new innovations, such as Continuous Flow Intermittent Cleaning (CFIC), have been developed to provide even more compact and efficient treatment processes [4]. The key distinction between MBBR and CFIC lies in their carrier filling fractions—the ratio of biocarrier volume to total reactor volume. CFIC systems typically have a filling fraction as high as 90 %, compared to MBBR systems, for which the filling fraction must generally does not exceed 70 % [5,11].

The biofilm carriers used in MBBR systems to support biofilm growth are typically made of high-density polyethylene (HDPE) and come in various dimensions, shapes, and surface areas. The first generation of biofilm carriers used in MBBR systems were the K-series made of polyethylene with a density of 0.95 g cm^−3^ [5,9]. A critical factor that differentiates biofilm carrier types is the available surface area for biofilm development. Specifically, the effective available surface area (i.e., m^2^/m^3^) refers to the protected inner portion of the carrier that remains free from contact with other carriers during mixing [5]. Microbial populations attach and proliferate on this protected surface area. However, the microbial diversity and composition on these carriers can be significantly influenced by the material properties of the carrier itself. Characteristics such as hydrophobicity and low surface energy—driven by apolar *Lifshitz-van der Waals forces* and *polar Lewis acid−base interactions*—play a key role in bacterial adhesion. These material properties may, in some cases, limit the attachment of microbial cells [12,13]. The selection of biofilm carriers varies depending on the treatment process, such as aerobic or anoxic/anaerobic systems. Carriers with wider openings are typically used for fast-growing aerobic heterotrophic biofilms, while those with smaller openings are preferred for nitrification and anammox processes. The surface properties of biofilm carriers can be modified through physicochemical methods to enhance bacterial attachment and biofilm formation [5,12,14].

Biofilms are complex, microbially diverse micro-ecosystems formed by the immobilization of bacteria on carrier surfaces [15,16]. Biofilm formation occurs naturally, beginning with cell attachment, followed by the development of microcolonies and secretion of extracellular polymeric substances (EPS). EPS play a critical role in biofilm development, aiding in the maturation of dense aggregates adhered to carrier surfaces. The protein-to-polysaccharide (PN/PS) ratio of EPS serves as an indicator of biofilm age, with a high protein ratio enhancing cell adhesion [5,[16], [17], [18]]. A dense biofilm eventually reaches a semi-steady state, balancing growth with detachment processes such as abrasion, erosion, sloughing, and grazing [5,19]. However, the microbial composition of biofilms on carriers also depends on substrate concentration gradients and the type of biological process (aerobic or anoxic). For example, in heterotrophic microbial communities, biofilm thickness is influenced by the diffusion of organics, nutrients, and oxygen. Microbial composition and diversity are further shaped by factors such as temperature, ammonium concentrations, pH, emerging wastewater contaminants, genetic regulatory mechanisms, and signaling pathways [19].

The MBBR process has demonstrated its effectiveness in nitrogen removal, even at cold and diluted wastewater temperatures as low as 5 °C, showing only a minor impact on nitrification and denitrification rates [20]. The efficiency of nitrogen removal in MBBR systems largely depends on the dominant microbial populations within the system and the biofilm thickness, particularly in facilitating simultaneous nitrification-denitrification (SND) [21,22]. Several studies have reported maximum total nitrogen removal efficiencies ranging between 86 % and 95 % in MBBR systems [[21], [22], [23]].

In addition to nitrogen removal, enhanced biological phosphorus removal (EBPR) has been successfully achieved in continuous multistage MBBR systems treating municipal wastewater [24]. However, implementing the EBPR process in MBBR systems poses challenges, as it requires alternating anaerobic and aerobic (AN/AE) conditions. Phosphorus removal in aerobic MBBRs has been achieved through cellular assimilation, such as in the treatment of cheese production wastewater. Additionally, phosphorus removal in continuous flow MBBR systems has been accomplished through chemical addition and subsequent precipitation [25].

To achieve optimal performance in MBBR and CFIC treatment processes, understanding biofilm characteristics—such as formation and thickness—and microbial diversity is crucial. Several studies have demonstrated that microbial community composition serves as a key indicator of robust biological process functionality, given the strong correlation between microbiome dynamics and system performance and stability [26]. Profiling bacterial composition in MBBR and CFIC wastewater treatment plants is therefore essential for efficient process control and for gaining deeper insights into the biological processes occurring within these systems.

In recent years, high-throughput DNA sequencing has emerged as the primary tool for studying microbial diversity [27]. However, limited information exists on microbial composition and dynamics in full-scale operational MBBR and CFIC biofilm reactors. This study aims to address this knowledge gap by investigating the microbial diversity and community dynamics in selected full-scale MBBR and CFIC plants in Norway. Specifically, the study examines four full-scale MBBR plants and two full-scale CFIC plants treating municipal and industrial wastewater. The microbial community diversity in these plants was analysed in relation to wastewater characteristics, biofilm carrier types, treatment technologies, and biological processes. Additionally, the study conducted a comparative analysis of microbial communities within treatment units of the same plant (beta diversity) as well as across different wastewater treatment plants (gamma diversity). This comparative approach was designed to identify similarities and differences in microbial diversity resulting from operational variations.

## 2 Results

### 2.1 Wastewater characteristics and constituents

The total chemical oxygen demand (tCOD), and soluble oxygen demand (sCOD) concentrations of wastewater samples collected from the MBBR plants in Tønsberg (TN), Arendal (AR), Monsrud (MN), and Holmestrand (HL) exhibited slight variations. The highest tCOD concentration, 514 mg/L, was observed in the TN plant's sample, while the lowest concentration, 250 mg/L, was recorded in the HL plant's sample. On average, the tCOD across all four municipal wastewater treatment plants was 386 mg/L (±120). In contrast, the tCOD concentrations in the Lindesnes (LN) and Bamble (NSO) plants were significantly higher, with values of 7737 mg/L and 4070 mg/L, respectively.

### 2.2 Biofilm growth and accumulation on the biofilm carriers

**Fig. 1:**
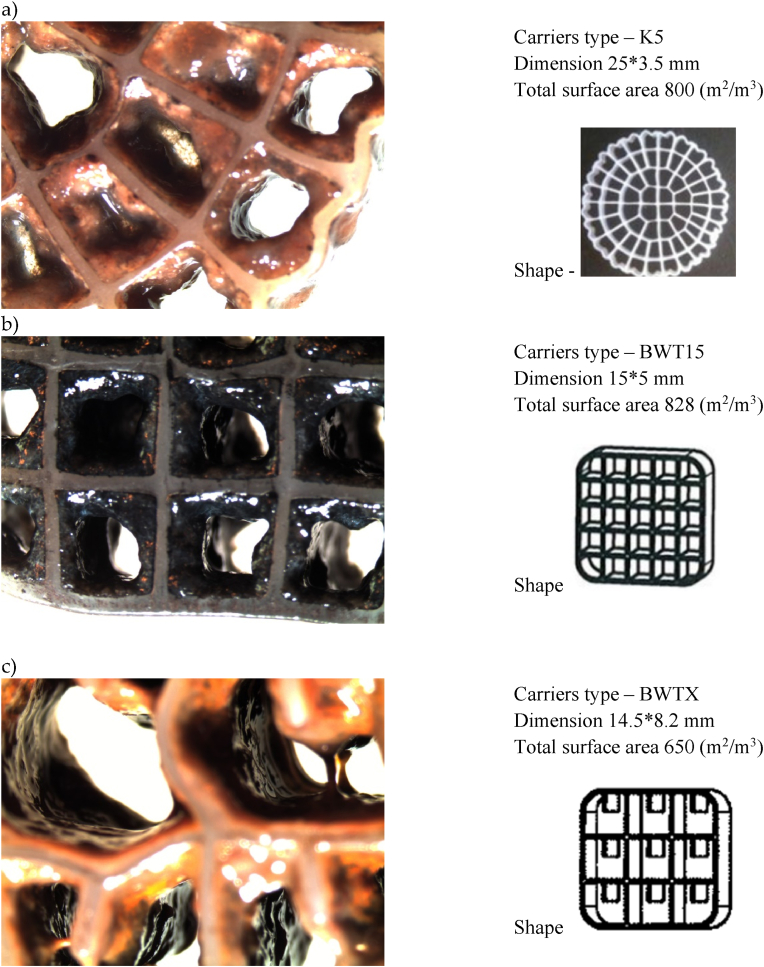
Microscopic images of biofilm formation on carrier cells from treatment plants—(a) Tønsberg (TN), (b) Arendal (AR), and (c) Monsrud (MN). The images were captured using a Nikon stereo microscope at 20 × magnification.

**Fig. 2:**
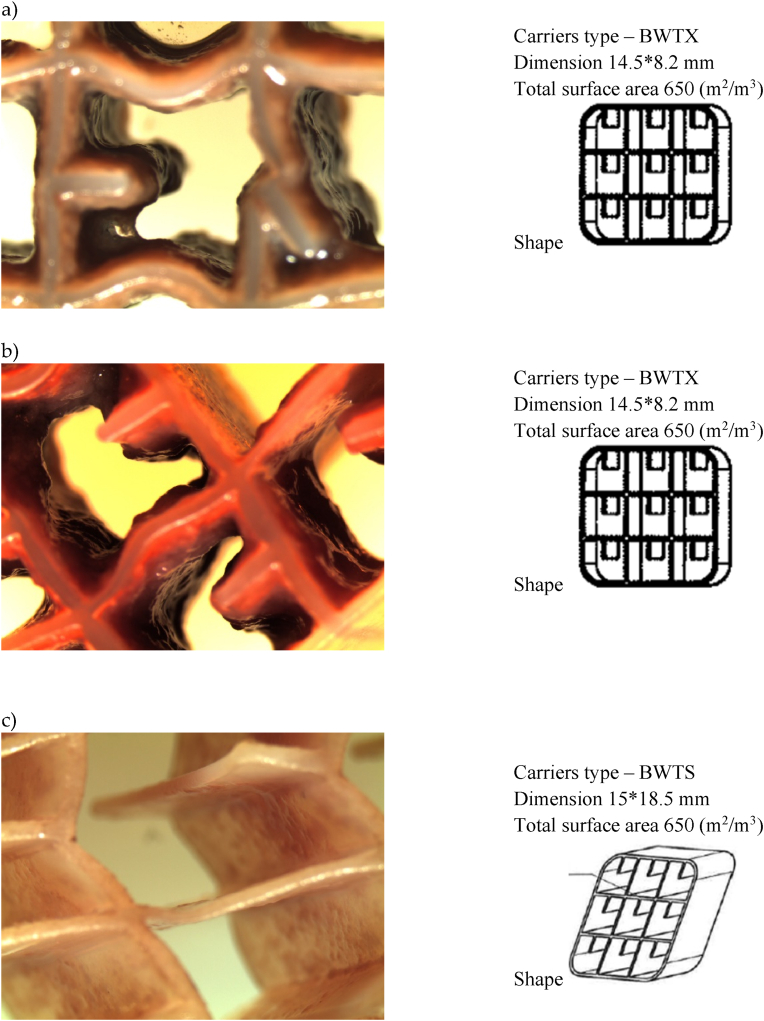
Microscopic images of biofilm formation on carrier cells from treatment plants—(a) Holmestrand (HL), (b) Lindesnes (LN), (c) Bamble (NSO). The images were captured using a Nikon stereo microscope at 20 × magnification.

The biofilm growth on the biofilm carriers was highly different between the biofilm carrier types, as seen under the microscopic observation (Fig. 1, Fig. 2a–c). The biofilm carriers K5 and BWT15 (Fig. 1a and b) were observed to have a very dense layer of biofilm growth in the carrier cells. The measured and calculated biomass weight per m^2^ of the biofilm carriers from the different MBBR and CFIC plants ranged from 7.2 g/m^2^ to 68.8 g/m^2^. The highest biomass accumulation was observed in the TN (Fig. 1a) and MN (Fig. 1c) MBBR plants, with values of 68.8 g/m^2^ and 22.6 g/m^2^, respectively. In contrast, the NSO CFIC plant (Fig. 2c), which treats industrial petrochemical wastewater using BWTS biofilm carriers, showed biomass accumulation ranging between 7.2 g/m^2^ and 17.4 g/m^2^.

### 2.3 Microbial sequencing in the MBBR biofilm reactors

**Fig. 3:**
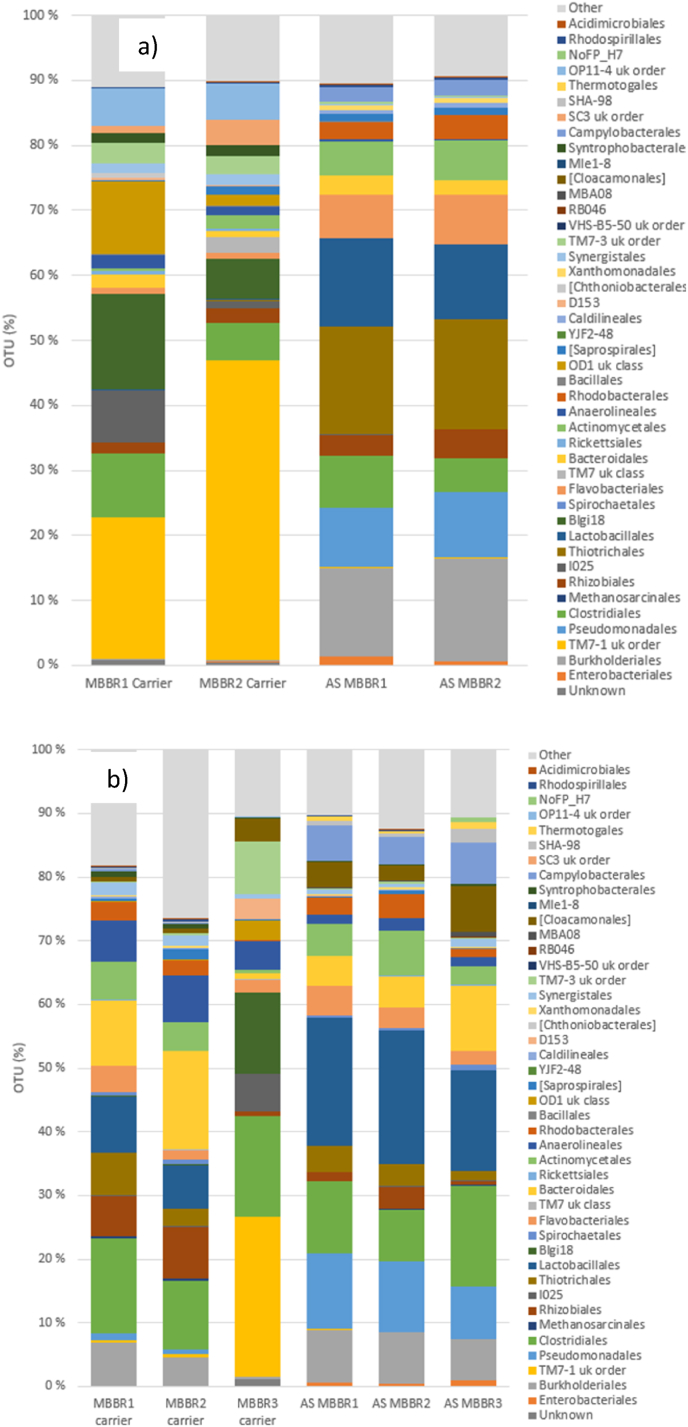
The microbial assemblage, represented as operational taxonomic units (OTUs) at the order level, was analysed on the biofilm carriers and in the liquid suspension a) at the Tønsberg MBBR plant and b) at Arendal MBBR plant.

The microbial genomic sequencing results from the Tønsberg WWTP revealed highly diverse bacterial orders present on the biofilm carriers and in the bulk liquid suspension (referred to as activated sludge, AS) from the two MBBR lines (MBBR1 and MBBR2) (Fig. 3a). Notably, the bacterial population on the biofilm carriers exhibited a completely distinct community structure compared to that in the liquid suspension samples. Furthermore, the fraction of dominant orders (measured as OTU %, operational taxonomic unit) on the biofilm carriers and in the liquid suspension showed slight variations between the two parallel MBBR lines, indicating beta species diversity. The biofilm carriers were dominated by bacterial orders TM7-1 (*Saccharibacteria*), Blgi18, and OP11-4, whereas the liquid suspension samples were predominantly composed of *Thiotrichales*, *Burkholderiales*, *Lactobacillales*, and *Pseudomonadales*.

Fig. 3b illustrates the microbial assemblage at the order level for the Arendal wastewater treatment plant. From a beta diversity perspective, the microbiome profiles of samples from the three reactors were generally similar, particularly between the two biofilm carrier samples collected from MBBR1 and MBBR2. However, the microbial community diversity on the biofilm carriers in MBBR3 showed slight variation, characterized by a higher proportion of the genus Saccharibacteria (TM7-1) alongside other bacterial communities. Across all biofilm carrier samples, the most dominant bacterial order was Clostridiales.

**Fig. 4:**
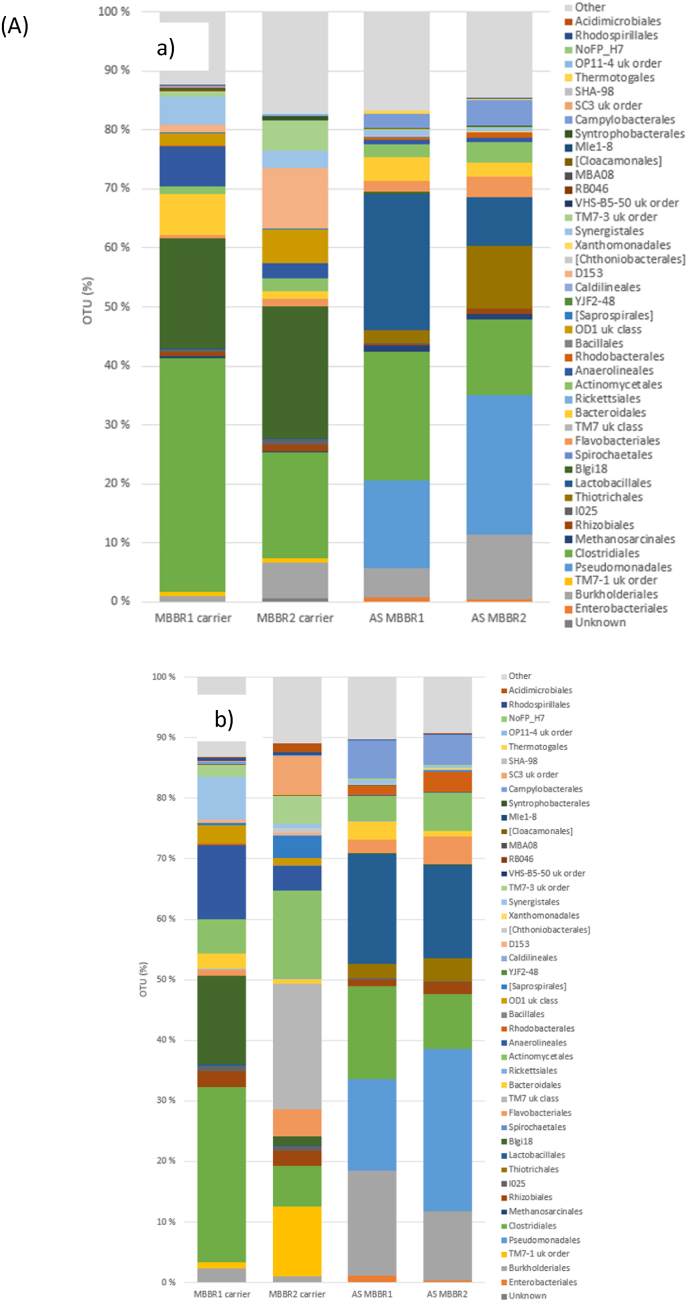
The microbial assemblage, represented as operational taxonomic units (OTUs) at the order level, was analysed on the biofilm carriers and in the liquid suspension a) at Monsrud MBBR plant and b) at Holmestrand MBBR plant.

The microbial assemblage at the order level for the Monsrud and Holmestrand wastewater treatment plant is shown in Fig. 4a. The most dominant orders on the biofilm carriers from Monsrud MBBRI and MBBRII (i.e. two parallel MBBRs) were Clostridiales and Blgi18. In the liquid suspension (i.e. activated sludge) samples, *Clostridiales* and *Pseudomonadales* were also dominant, though in varying proportions. Additionally, *Lactobacillales* and *Thiotrichales* were among the most prevalent bacterial communities observed in the liquid suspension samples from both reactors, highlighting beta diversity.

In the Holmestrand MBBR plant, the dominant bacterial orders in MBBRI were *Clostridiales*, Blgi18, and *Anaerolineales*, while in MBBRII, TM7, *Actinomycetales*, and TM7-1 were predominant. In the liquid suspension (AS) from MBBRI, the dominant orders included *Lactobacillales*, Burkholderiales, Clostridiales, and *Pseudomonadales*. Similarly, in liquid suspension (AS) from MBBRII, the most prevalent orders were *Pseudomonadales*, *Lactobacillales*, *Burkholderiales*, and *Clostridiales* (Fig. 4b).

### 2.4 Microbial sequencing in the CFIC biofilm reactors

**Fig. 5:**
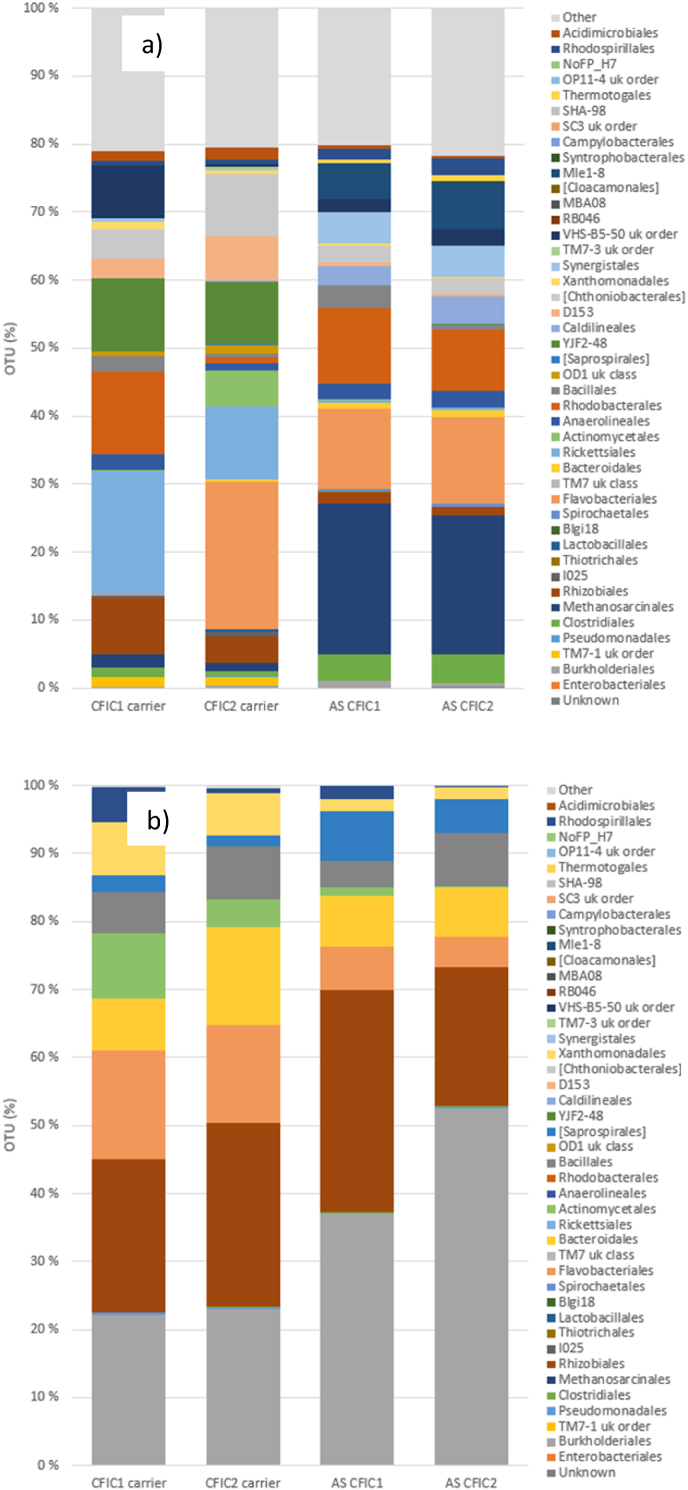
The microbial assemblage as operational taxonomic units (OTU) at the order level from biofilm carriers and liquid suspension (AS) a) from Lindesnes CFIC plant and b) from NSO CFIC plant.

In the Lindesnes plant, the bacterial community profiles on the biocarriers across the two reactor lines (CFIC1 and CFIC2) exhibited high diversity (Fig. 5a). On the biocarriers from CFIC1, the most dominant bacterial orders were *Rickettsiales*, *Rhodobacterales*, and *Rhizobiales*, whereas in CFIC2, *Flavobacteriales*, *Rickettsiales*, *Actinomycetales*, and *Rhizobiales* were predominant. In contrast, the bacterial orders and their relative abundances in the liquid suspension (activated sludge, AS) differed significantly from those observed on the biocarriers. For instance, the dominant bacterial orders in the liquid suspension (AS) were *Methanosarcinales*, *Flavobacteriales*, *Rhodobacterales*, and *Clostridiales*.

The biological samples from the biofilm carriers and liquid suspensions (activated sludge, AS) in the CFIC reactors of the NSO plant exhibited similar microbial genera, with slight differences in abundance. At the order level, the most dominant microbial communities were *Burkholderiales*, *Rhizobiales*, *Flavobacteriales*, and *Bacteroidales*. Additionally, other bacterial orders, such as *Actinomycetales*, *Bacillales*, and *Xanthomonadales*, were also prevalent in both the biofilm carriers and liquid suspension samples (Fig. 5b).

### 2.5 Hierarchical clustering of the microbiome in the MBBR and CFIC biofilm reactors

**Fig. 6:**
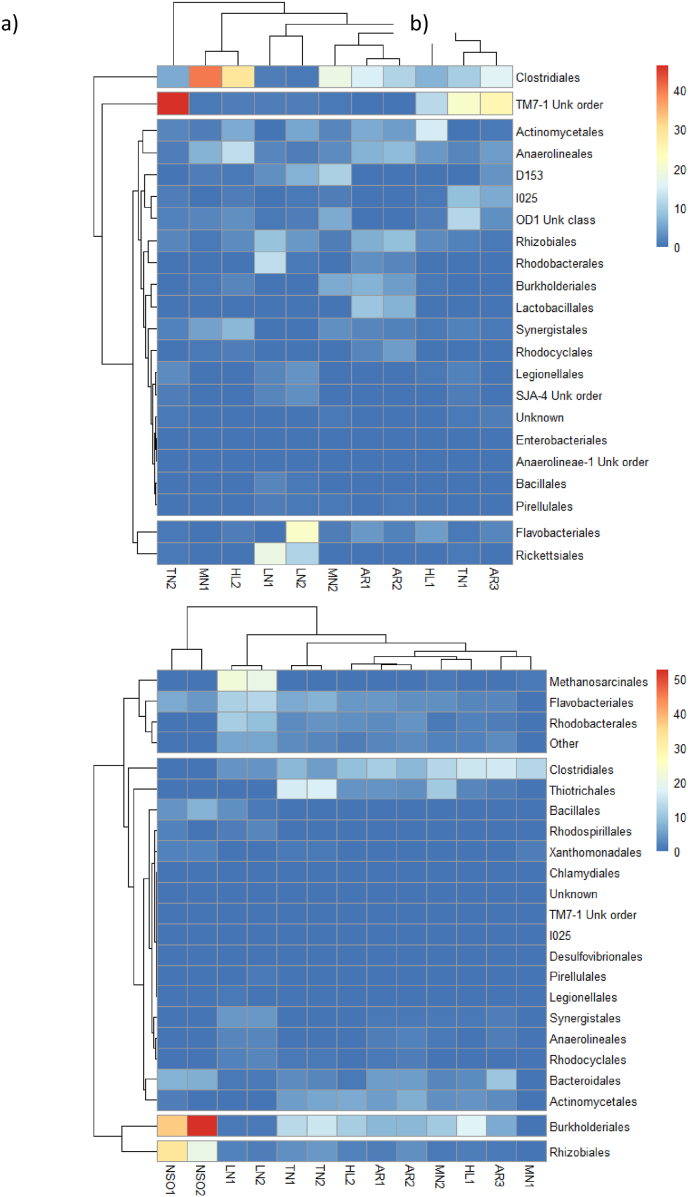
Hierarchical clustering of microbial genera at the order level was performed for biofilm carriers a) and liquid suspension b) from the MBBR and CFIC biofilm reactors. The treatment plants included Arendal (AR), Holmestrand (HL), Lindesnes (LN), Monsrud (MN), and Tønsberg (TN). The numbers represent the corresponding reactor numbers.

A heatmap with hierarchical clustering of microbiome genera on the biofilm carriers from different treatment plants was used to visualize the dominant genera across the studied treatment plants (Fig. 6a). This approach helps to understand the microbiome at a gamma-level diversity among the various MBBR and CFIC plants targeted in this study. The hierarchical clustering groups similar observations close to one another, with the color gradient indicating the relative abundance of each order across the treatment plants. For example, the orders TM7-1 and *Clostridiales* were found in higher proportions on biofilm carrier samples from the Tønsberg and Monsrud treatment plants (Fig. 6a and b). In contrast, the microbial genera in the liquid suspension (activated sludge) differed from those on the biofilm carriers. The most dominant genera in the liquid suspension were *Burkholderiales* and *Rhizobiales*, which were shown to be particularly abundant in the NSO treatment plant (Fig. 6b).

**Fig. 7:**
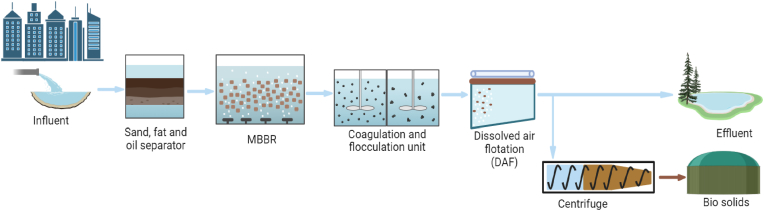
A schematic flow diagram of the full-scale wastewater treatment plants in Tønsberg (TN), Arendal (AR), Monsrud (MN), and Holmestrand (HL). The treatment process consists of three main stages: mechanical, biological (MBBR), and chemical treatment units. This multi-stage process is designed to effectively treat wastewater by removing organics and recovering valuable biosolids.

## 3 Discussion

### 3.1 Wastewater characteristics of the plants

Municipal wastewater can be classified as strong, medium, or weak based on the concentration of its major organic and inorganic constituents. Wastewater with a total chemical oxygen demand (tCOD) in the range of 250–500 mg/L is categorized as weak to medium [28,29]. In this study, the wastewater characteristics of the Arendal, Holmestrand, Monsrud, and Tønsberg treatment plants fall within this category. These plants also display similar profiles for organic carbon and inorganic nutrients (i.e. nitrogen and phosphorus). The average annual tCOD removal efficiency of the Tønsberg wastewater treatment plant is 86 % (other treatment plants data not shown). In contrast, the wastewater in the Lindesnes and NSO treatment plants is classified as strong, with a tCOD exceeding 1000 mg/L, as these plants primarily treat wastewater originating from medical and industrial sources, respectively [28,30,31].

The concentration of organic and inorganic constituents, along with the presence of toxic compounds in wastewater, plays a key role in shaping the structure and diversity of the microbiome community within a system. In biological wastewater treatment processes, microbes transform these organic and inorganic components into biomass and various chemical forms that can be easily separated from the wastewater. Consequently, the characteristics of the wastewater, the treatment process, and the type of reactor used can influence microbial composition and diversity. Study show that in short timescales wastewater physicochemical characteristics explained 61 % of the total microbial community variance [32]. For instance, variations in microbiome diversity observed between MBBR and CFIC biofilm reactors may be attributed to differences in wastewater characteristics, reactor configurations and operational characteristics. For instance, studies have shown that variations in the concentration of ammonia nitrogen in the influent significantly affect the composition of the microbial community in MBBR systems [33]. Operational parameters such as temperature, pH, and specific operating conditions have been shown in numerous studies to significantly impact microbial community assemblages in biological wastewater treatment systems [[34], [35], [36], [37], [38]].

### 3.2 Biofilm growth on biofilm carriers

In MBBR and other fixed-biofilm reactors, biofilm carriers are specifically designed or selected based on the reactor type and the objectives of the wastewater treatment process. The choice of biofilm carriers in these reactors depends on their capacity to retain biomass and their ability to achieve the desired treatment efficiency [5,6]. This study utilized four distinct types of biofilm carriers K5, BWT15, BWTS, and BWTX in MBBR and CFIC wastewater treatment plants (Table 1). These carriers varied in dimensions, number of cells, and total protected surface area. By increasing the biofilm carrier fill ratio (i.e., the volume of biofilm carriers relative to the total volume of the reactor), the total available surface area within the reactor can be expanded. This, in turn, enhances the treatment efficiency of these bioreactors [6,39]. For instance, in MBBR reactors, the efficiency of the nitrification process is heavily influenced by the type of biofilm carrier utilized [40].

**Table 1:** Characteristics of the biofilm carriers used in the six full scale MBBR and CFIC wastewater treatment plants.

Plant name	WW* source	Biofilm reactors type	Biofilm carriers Type	Biofilm carriers dimension (L x B* mm)	Biofilm carriers total surface area (m^2^/m^3^)	Biofilm carriers Shape
Tønsberg (TN)	Municipal	MBBR	K5	25 × 3.5	800	
Arendal (AR)	Municipal	MBBR	BWT15	15 × 5	828	
Monsrud (MN)	Municipal	MBBR	BWTX®	14.5 × 8.2	650	
Holmestrand (HL)	Municipal	MBBR	BWTX®	14.5 × 8.2	650	
Lindesnes (LN)	Medical	CFIC®	BWTX®	14.5 × 8.2	650	
NSO Bamble (NSO)	Industry	CFIC®	BWTS®	15 × 18.5	650	

All the biofilm carriers used in this study were made of high-density polyethylene (HDPE), a material known for its supportive properties, including hydrophobicity and low surface energy, which enhance cell attachment. A more hydrophilic surface results in a higher biofilm formation rate and increased biofilm thickness on the media surface. Hydrophilic materials like as Polyethylene terephthalate (PET) and HDPE have shown superior performance in wastewater treatment [12,41]. In our investigation, biofilm growth on the carriers varied (Fig. 1, Fig. 2a–c), likely influenced by the type of biofilm carriers, reactor design, and treatment process conditions. Key factors such as the size, shape, and surface area-to-volume ratio of the carriers significantly affect biomass accumulation. For example, the K5 biofilm carriers exhibited substantial biofilm growth and biomass thickness (Fig. 1a). Additionally, biomass accumulation depends on microbial growth kinetics and the frequency of carrier washing. For instance, the NSO treatment plant employs CFIC technology, which features a forward flow cleaning mechanism to periodically remove excess biomass from the carriers (Fig. 2c). Biofilm thickness depends on various factors, including organic loading, shear forces, temperature, and oxygen concentration. Additionally, biofilm thickness can be influenced by the type of media used and the loading rate. Under aerobic conditions, it is preferable to maintain biofilm thickness below 150 μm. Research has also shown that thinner biofilms (20–30 μm) are associated with higher nitrification rates. Overall, biofilm thickness plays a crucial role in determining the efficiency of MBBR systems and nutrient removal processes [[42], [43], [44], [45]].

The formation of biofilms on biofilm carriers begins with the attachment of multiple cells, followed by bacterial growth that develops into a complex, heterogeneous microbial aggregate or mature biofilm. During this process, extracellular polymeric substances (EPS), biopolymers produced by cells play a crucial role in facilitating bacterial attachment to the carriers. The chemical composition of EPS is highly complex, with polysaccharides, proteins, nucleic acids, and lipids as its major components. The secretion of EPS, driven by the microbial community and its metabolism, is closely linked to biofilm thickness. Furthermore, EPS play a critical role in providing stability and promoting the adherence of biofilms to carriers in MBBR systems [[46], [47], [48], [49]].

Factors such as nutrient availability, pH, temperature, and water hydrodynamics significantly influence biofilm formation [16,41,47]. Additionally, positively charged surfaces promote microorganism growth. A positively charged biocarrier surface (∼7.80 mV) enhances bacterial attachment, whereas negatively charged surfaces limit adhesion rates and reduce the stability of attached biomass [50]. Furthermore, the geometric properties of the carriers significantly affect biofilm thickness. Previous studies have shown that certain carrier designs result in lower biofilm thickness and density per reactor volume [51]. These findings highlight the strong relationship between the type of biofilm carrier used in reactors, biofilm thickness, and the diversity of microbiomes within the treatment system.

### 3.3 Microbial sequencing in MBBR and CFIC reactors

Microbial sequencing analysis of biofilms grown on carriers in the MBBR and CFIC reactors revealed that both the treatment process and carrier type directly influenced the microbial community structure. For example, biofilm carriers at the Tønsberg MBBR plant were predominantly colonized by the genus TM7, belonging to the phylum *Saccharibacteria*. This candidate phylum, formerly known as TM7, is abundant in the activated sludge of wastewater treatment plants and plays a crucial role in degrading various organic compounds under both aerobic and anaerobic conditions [52]. Moreover, in most activated sludge reactors treating domestic wastewater, the order *Burkholderiales* is predominantly abundantly [53]. The order *Burkholderiales* includes the family Alcaligenaceae, which is capable of simultaneous heterotrophic nitrification and denitrification in MBBR systems treating rejected water (water after sludge dewatering) [54]. *Burkholderiales* have also been reported as the dominant order in activated sludge process treating polycyclic aromatic hydrocarbons (PAHs) [55]. These diverse bacterial orders comprise several families of bacteria species found in wastewater activated sludge [56,57].

Our study revealed a significant difference in the microbial community structure between the biofilm carriers in MBBR reactors and the liquid suspension (Fig. 3a and b). Previous studies have reported successional changes in microbial communities as biofilms develop in full-scale MBBR systems treating municipal wastewater [10,58]. Interestingly, while the order *Clostridiales* is typically considered anaerobic, it has also been observed in aerobic MBBR systems, likely due to community shifts over time [10,59]. These microbial groups are well known for their role in the removal of organic and nutrients, including nitrogen compounds [10,60].

The microbial diversity in the Lindesnes CFIC biofilm reactor was distinct, influenced by the treatment plant configuration and the characteristics of the wastewater. The predominant orders on the biofilm carriers were *Rickettsiales*, *Flavobacteriales*, and *Rhodobacterales*. *Rickettsiales* are known to inhabit protists, such as ciliates and amoebae, and include many pathogenic microbes in symbiotic associations [61]. This is likely because, unlike typical municipal wastewater, hospital wastewater often contains infectious microbes and pharmaceuticals [62]. Additionally, the presence of anaerobic bacteria, such as *Methanosarcinales*, in the liquid suspension of the CFIC biofilm reactor at the Lindesnes plant may be attributed to the plant's anaerobic digestion (AD) unit. Effluent from the AD unit is recycled for further treatment, contributing to this microbial diversity. Among archaea, genera such as *Methanosaeta*, *Methanobacterium*, and families like *Methanosarcinaceae* are commonly found in high relative abundance in anaerobic digesters [63]. In contrast, the NSO plant treated petrochemical wastewater originating from the washing water of oil tanks. The predominant order in this treatment plant was *Burkholderiales*, as confirmed by the hierarchical clustering heat map, which shows *Burkholderiales* as dominant, followed by *Rhizobiales* (Fig. 6b). A study revealed that *Burkholderiales* is known for degrading aromatic organic compounds such as phenol, benzoate, toluene, naphthalene, and phenanthrene [64]. Moreover, *Rhizobiales* and *Flavobacteriales* were subdominant in the NSO CFIC biofilm reactor. Previous studies have reported that these orders play a significant role in degrading aromatic pollutants, which aligns with their presence in the NSO CFIC plant [64,65].

This study highlights a distinct microbial community structure present on the biofilm carriers and in the liquid suspensions of MBBR and CFIC reactors. Notably, in biofilm-based wastewater treatment processes, biofilm carriers offer significant advantages over conventional activated sludge (AS) methods. These benefits stem from the higher microbial diversity supported by the protected surfaces of the biofilm carriers [41]. Furthermore, the findings of this study reinforce the well-established observation that the microbiome diversity on biofilm carriers surpasses that of the microorganisms found in suspension within the liquid phase of biofilm reactors.

## 4 Conclusions

MBBR and CFIC biofilm reactors are compact and highly efficient wastewater treatment systems. The biofilm carriers used in these reactors possess distinct properties, including shape, dimensions, and total surface area, which facilitate the attachment and growth of diverse microbial populations on their surfaces. In addition to the biofilm-attached microorganisms, a significant number of microbes are present in the liquid suspension as activated biomass in MBBR and CFIC systems. Microbiome sequencing and taxonomic classification revealed significant diversity in bacterial community composition, structure, and abundance across different treatment plants. This diversity was influenced by several factors, including wastewater physicochemical characteristics, the type and properties of biofilm carriers, operating conditions, and the reactor technology employed. In conclusion, the most dominant bacterial populations on the biofilm carriers across all treatment plants belonged to the orders TM7-1 (*Saccharibacteria*), *Burkholderiales*, *Clostridiales*, *Actinomycetales*, *Pseudomonadales*, *Rickettsiales*, and *Rhodobacterales*. Conversely, the dominant microbial orders in the liquid suspension (active biomass) were *Clostridiales*, *Methanosarcinales*, *Pseudomonadales*, *Flavobacteriales*, and *Rhodobacterales*. The microbial diversity observed on the biofilm carriers was remarkably high. Additionally, the combined microbial diversity on both the biofilm carriers and in the liquid suspension provided complementary advantages, contributing to the effective removal of organic matter, nutrients, and toxic contaminants from wastewater in these compact MBBR and CFIC biofilm reactors.

## 5 Materials and methods

### 5.1 MBBR and CFIC wastewater treatment plants

The treatment plants located in Tønsberg (TN), Arendal (AR), Monsrud (MN), and Holmestrand (HL) were of the MBBR type, while the plants in Lindesnes (LN) and NSO Bamble (NSO) utilized CFIC biofilm reactors. Table 1 provides an overview of the wastewater type, biofilm carrier characteristics, and the shapes of the biofilm carriers used in all the full-scale plants. The reactors at the Monsrud, Holmestrand, and Lindesnes plants employ the same BWTX carriers, which have a total surface area of 650 m^2^/m^3^ and cell dimensions of 14.5 mm × 8.2 mm. In contrast, the Tønsberg, Arendal, and NSO Bamble plants each use different carrier types namely K5, BWT15, and BWTS with total surface areas of 800, 828, and 650 m^2^/m^3^, respectively (Table 1). It is well established in the literature that all these carrier types create a favorable microenvironment for microbial growth due to their high degrees of inter-compartmentalization. The biological unit processes at all six treatment plants are designed to achieve the removal of biological oxygen demand (BOD) and total solids (TS).

The four treatment plants (TN, AR, MN, HL) have similar process flows and treatment units, as illustrated in Fig. 7. The treatment process consists of three main stages: i) *Mechanical process unit* to remove sand, fat, and oil, ii) *Biological process unit (MBBR)* to remove biological oxygen demand (BOD) and iii) *Chemical process unit* to remove total solids (TS). These plants primarily treat municipal wastewater from their respective cities. The biological process units at TN, MN, and HL operate with two parallel MBBR lines (referred to as MBBR1 and MBBR2), with each line containing two biofilm reactors arranged in sequence. In contrast, the AR treatment plant features three parallel MBBR lines (designated as MBBR1, MBBR2, and MBBR3).

**Fig. 8:**
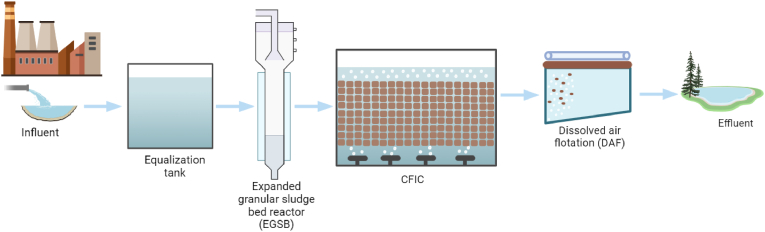
A schematic flow diagram of Lindesnes (LN) wastewater treatment plant. The LN plant has an expanded granular sludge bed reactor (EGSB), which is designed to reduce the organic load entering the CFIC biofilm reactors.

**Fig. 9:**
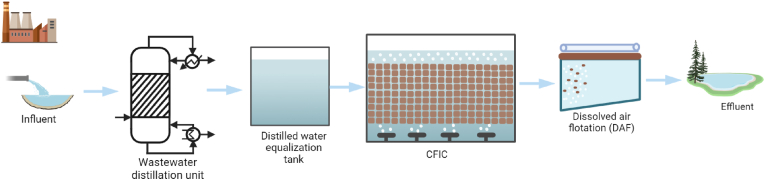
A schematic flow diagram of NSO Bamble (NSO) wastewater treatment plant. The NSO plant features a preliminary treatment stage that includes a wastewater distillation unit to designed to remove oil before the wastewater enters the equalisation tank.

The treatment plants at Lindesnes (LN) and NSO Bamble (NSO) are CFIC biofilm reactors designed to treat specific types of industrial wastewater. The LN plant handles wastewater from the pharmaceutical industry, while the NSO plant processes petrochemical wastewater, including effluents from manufacturing industries, auto repair shops, and oil tank washing. The treatment process flows and unit configurations for the LN and NSO facilities are illustrated in Fig. 8, Fig. 9, respectively.

Both plants employ the innovative CFIC biofilm process, a compact and efficient treatment method. This process is specifically designed to support biofilm growth under variable load conditions and in toxic wastewater environments. Each plant operates two parallel lines of CFIC process units to enhance efficiency. Additionally, the LN plant includes two biological expanded granular sludge bed (EGSB) reactors, which operate in parallel to reduce the organic load on the CFIC units.

### 5.2 Sampling and physiochemical characterization of wastewater

The sampling and physicochemical analysis of wastewater from four full-scale MBBR and two full-scale CFIC biofilm reactors were conducted over different periods, specifically from September 7 to October 28, 2022. Samples were collected from each reactor and analysed following the standard methods for the examination of water and wastewater [66]. The physiochemical wastewater analysis was conducted for total and soluble chemical oxygen demand (tCOD and sCOD), ammonium (NH_4_^+^), nitrate (NO_3_^−^), nitrite (NO_2_^−^), total solid (TS), volatile solid (VS) and alkalinity. For all analyses except tCOD measurement, samples were filtered using 0.45 μm GxF multi-layered acrodisc PSF filters. Prior to tCOD analysis, the samples were homogenized. Spectro-quant test cells with appropriate measuring ranges were used for all tests.

For all measurements, the Spectro-quant® Prove 100 (Merck KGaA, Darmstadt, Germany) was utilized. The ammonium, nitrite, and nitrate cell tests correspond to APHA 4500-NH3 F, APHA 4500-NO_2_-, and APHA 4500-NO_3_-, respectively. The chemical oxygen demand (COD) was assessed using a method analogous to APHA 5220.

The wastewater pH was measured using a Hanna Instruments HI-5222-02 pH/ISE/ORP meter (MT00121, Kungsbacka, Sweden). Calibration of the pH meter was performed before each measurement using standard buffer solutions at pH 4.0, 7.0, and 9.0 to ensure precise readings. Alkalinity was determined using an alkalinity cell test. This involved pipetting 4.0 mL of Reagent AC-1 into a clean 16 mm round cell, followed by the addition of 0.5 mL of Reagent AC-2. The alkalinity value was then measured using the Spectro-quant® Prove 100 photometer at a wavelength of 605 nm. As the photometer displayed results in mmol/L, the alkalinity value was converted to mg/L CaCO3 by multiplying the reading by 50.04.

The total solids (TS) and volatile solids (VS) were measured in accordance with APHA Standard Methods 2540 D and 2540 E, respectively.

### 5.3 Active biomass on the biofilm carriers and biofilm morphology observations

The biomass weight per square meter of biofilm carriers was measured using samples collected from each biofilm reactor. Initially, the biofilm carriers were dried at 105 °C for at least 2 h to remove any moisture. Following this, the biofilm carriers were weighed to record their initial weight, which included the biomass. These carriers were then washed thoroughly using a sodium hypochlorite (NaClO) solution and rinsed with tap water to remove the biofilm. After cleaning, the biofilm carriers were dried again at 105 °C for 24 h and weighed to determine their weight without biomass. The biomass per carrier was calculated as the difference between the weight of the biofilm carriers before and after washing, divided by the number of sampled biofilm carriers. This provided the average biomass weight for a single carrier (Eq. (1)).

Eq. 1 Biomass per biofilm carrier, (m) = (m_1_-m_2_)/N

Where, m_1_ is dried biofilm carriers, m_2_ dried biofilm carriers after washing and cleaning with sodium hypochlorite (NaClO) solution and tap water and N is the number of biofilm carriers.

The biomass weight per square meters (m^2^) was estimated based on the biomass per biofilm carriers multiplied by the number of biofilm carriers per cubic meter (m^3^) divided by the biofilm carrier protected surface area (Eq. (2)).

The biomass per unit protected surface area was calculated as:

Eq. 2 W = m * Vc/A

Where, W- Biomass per unit protected surface area (g/m^2^) m = Biomass per biofilm carrier (g/piece), Vc = Number of biofilm carrier pieces per m^3^ volume (pieces/m^3^). Based on dimensions, 1-m cube BWT15 (Biowater Technology AS) biofilm carrier contains 600,000 pieces and BWTX/BWTS (Biowater Technology AS) contains 400,000 pieces [67]. A = Total surface area (m^2^/m^3^).

(*WW = Wastewater, *L = Length; B = Breadth)

The biomass morphology on the biofilm carriers was observed using a Nikon Stereo microscope (SMZ745/SMz745T). The magnification was set to 20X, and the infinity analyze capture software version 6.5 (© 2023 Teledyne Lumenera) was used to capture and process the images.

### 5.4 Microbiome sampling and DNA extraction

Microbiome samples for DNA analysis were collected following a standardized protocol designed for sampling microbiomes from water and wastewater [68]. Prior to sampling, all treatment plants were in regular operation to ensure representative conditions. Biofilm carriers and liquid suspensions were collected in sterile 50 mL centrifuge tubes to prevent contamination. Parallel samples were collected from both biofilm carriers and liquid suspensions. Ice boxes were used for the collection and transportation of samples to maintain sample integrity during transit to the laboratory. For the liquid suspension samples, centrifugation was performed at 5000 rpm for 15 min to separate the solid phase. After centrifugation, the supernatant was carefully discarded to isolate the pellet containing microbial biomass. The solid pellet was immediately stored at −20 °C until further DNA extraction and analysis.

For DNA extraction from the biofilm carriers, each carrier was transferred into a 50 ml tube containing 3 ml sterile PBS solution and treated with ultrasound for 5 min. The biofilm carrier was then removed from the tube, and the slurry was centrifuged at 3900 g for 10 min. DNA was extracted from both the pellet and the supernatant and combined before sequencing. DNA was extracted from approximately 0.3 ml sample material using the Quick DNA Fecal/Soil Microbe Miniprep Kit (Zymo Research). DNA extraction of all the samples took five weeks.

### 5.5 Barcode sequencing and taxonomic classification

DNA was subjected to targeted amplicon sequencing of the V3+V4 region of the 16S ribosomal RNA gene. Sequencing libraries were generated following the Illumina “16S Metagenomic Sequencing Library Preparation guide” with the primers Bakt_341F and Bakt_805R [69] complemented with Illumina sequencing adapters (5′-TCGTCGGCAGCG TCAGATGTGTATAAGAGACAG-CCTACGGGNGGCWGCAG-3′ and 5′-GTCTCGTGGGCTCGGAGATGT GTATAAGAGACAG-GACTACHVGGGTATCTAATC C-3′). PCR products were purified by AMPure XP Agencourt (Beckman-Coulter) and quantified on a Qubit v2 using the Qubit dsDNA BR Assay Kit (Thermo Fisher Scientific). DNA libraries were pooled and sequenced on a MiSeq sequencer (Illumina) using the MiSeq reagent Kit v3 in the 2*300 bp paired-end mode. Sequencing reads were demultiplexed in Local Run Manager (Illumina), and further data processing was performed in CLC Genomic workbench v.21.0.2 (Qiagen). Sequence reads were adapter trimmed, filtered and Operational Taxonomic Units (OTU) were classified using the Data QC and OTU clustering workflow of the Microbial Genomics Module.

Bioinformatics analyses were performed in CLC Genomics Workbench 22.0 (Qiagen, Aarhus, Denmark) by running the CLC Microbial Genomics Module 22.1 with standard settings. Raw sequencing reads were trimmed for adapters and quality filtered using the “Trim Reads 2.6” tool. Trimmed reads were then filtered based on the number of reads discarding samples with <1 % median abundance using “Filter Samples Based on number of Reads 1.04”. Operational Taxonomic Units (OTUs) were called by the “OTU Clustering 2.6” tool in the Reference-based OTU clustering mode using the Greengenes Database version 13.5 with 97 % similarity for classification. Finally, tables were then processed in Microsoft Excel.

### 5.6 Data analysis

Further data processing was performed in Microsoft Excel. Due to the high diversity level of the sample, sequencing data is shown at the order level. To visualize hierarchical clustering a heat map is created using R-statistical program (version 4.3.1) in the pretty heatmap called ‘pheatmap’ package. In pheatmap R-package clustering was done by putting comparable rows or columns together based on the data matrix. The pheatmap includes clustering, scaling and annotation.

## CRediT authorship contribution statement

**Eshetu Janka:** Writing – review & editing, Writing – original draft, Software, Resources, Project administration, Methodology, Funding acquisition, Formal analysis, Data curation, Conceptualization. **Ram Prasath Alagappan:** Writing – review & editing, Methodology, Investigation, Formal analysis, Data curation. **Dipaluk Das:** Writing – review & editing, Methodology, Data curation. **Leif Arne Kjeldsberg:** Writing – review & editing, Resources, Data curation. **Shuai Wang:** Writing – review & editing, Methodology, Funding acquisition, Data curation, Conceptualization. **Tone Haugen:** Writing – review & editing, Software, Resources, Methodology, Formal analysis, Data curation. **Alexander Wentzel:** Writing – review & editing, Methodology, Funding acquisition, Conceptualization.

## Funding information

This research was funded by Vestfold and Telemark regional research fund, Norway. Project number 332865. Project title ‘*Biofilms characterization for wastewater treatment*’.

## Declaration of competing interest

The authors declare that they have no known competing financial interests or personal relationships that could have appeared to influence the work reported in this paper.

The author is an Editorial Board Member/Editor-in-Chief/Associate Editor/Guest Editor for this journal and was not involved in the editorial review or the decision to publish this article.

## Data Availability

The microbial genomic analysis data of this study, operating and removal data of the treatment plants are available on request.
